# Breast milk nutrient content and infancy growth

**DOI:** 10.1111/apa.13362

**Published:** 2016-04-06

**Authors:** Philippa Prentice, Ken K. Ong, Marieke H. Schoemaker, Eric A. F. van Tol, Jacques Vervoort, Ieuan A. Hughes, Carlo L. Acerini, David B. Dunger

**Affiliations:** ^1^ Department of PaediatricsMRL Wellcome Trust‐MRC Institute of Metabolic ScienceNIHR Cambridge Comprehensive Biomedical Research CentreUniversity of CambridgeCambridgeUK; ^2^ MRC Epidemiology UnitUniversity of CambridgeCambridgeUK; ^3^ Mead Johnson Pediatric Nutrition InstituteNijmegenNetherlands; ^4^ Wageningen UniversityWageningenNetherlands

**Keywords:** Breast milk, Growth, Macronutrients, Nutrition, Weight

## Abstract

**Aim:**

Benefits of human breast milk (HM) in avoiding rapid infancy weight gain and later obesity could relate to its nutrient content. We tested the hypothesis that differential HM total calorie content (TCC) or macronutrient contents may be associated with infancy growth.

**Methods:**

HM hindmilk samples were collected at ages 4–8 weeks from 614 mothers participating in a representative birth cohort, with repeated infancy anthropometry. HM triglyceride (fat), lipid analytes and lactose (carbohydrate) were measured by ^1^H‐NMR, and protein content by the Dumas method. TCC and %macronutrients were determined.

**Results:**

In 614 HM samples, fat content was as follows: [median(IQR)]: 2.6 (1.7–3.6) g/100 mL, carbohydrate: 8.6 (8.2–8.8) g/100 mL, protein: 1.2 (1.1–1.2) g/100 mL; TCC: 61.8 (53.7–71.3) kcal/100 mL. HM of mothers exclusively breast feeding vs. mixed feeding was more calorific with higher %fat, lower %carbohydrate and lower %protein. Higher HM TCC was associated with lower 12‐months body mass index (BMI)/adiposity, and lower 3–12 months gains in weight/BMI. HM %fat was inversely related to 3–12 months gains in weight, BMI and adiposity, whereas %carbohydrate was positively related to these measures. HM %protein was positively related to 12‐months BMI.

**Conclusion:**

HM analysis showed wide variation in %macronutrients. Although data on milk intakes were unavailable, our findings suggest functional relevance of HM milk composition to infant growth.

**Abbreviations**
ALA: Alpha‐linolenic acid
BMI: Body mass index
CBGS: Cambridge Baby Growth Study
DHA: Docosahexaenoic acid
HM: Human breast milk
IQR: Interquartile range
LC‐PUFA: Long‐chain polyunsaturated fatty acid
NMR: Nuclear magnetic resonance
NOESY: Nuclear Overhauser effect spectroscopy
SDS: Standard deviation score
TCC: Total calorie content
WHO: World Health Organisation

**Key notes**

- Breast feeding is associated with lower rates of infancy weight gain and later obesity; however, data on breast milk composition and relationships with growth are sparse.
- Macronutrient contents of 614 hindmilk samples were as follows: fat: [median(IQR)]: 2.6 (1.7–3.6) g/100 mL, carbohydrate: 8.6 (8.2–8.8) g/100 mL and protein: 1.2 (1.1–1.2) g/100 mL.
- %Carbohydrate was positively related and %fat inversely related to later infant weight and adiposity; %protein was positively related to body mass index; suggesting functional implications of breast milk macronutrient contents.

## Introduction

It is increasingly recognised that early postnatal nutrition, as well as being critical for optimal infancy growth, may also be associated with long‐term health outcomes 1. Rapid early infant weight gain predisposes to an adverse metabolic phenotype in later life, with increased risk of overweight 2, central adiposity and insulin resistance 3. The type of infant milk feeding (exclusive breast feeding, formula feeding or mixing feeding), as well as specific dietary compositions and volume of intake, may be important factors.

In contemporary Western settings, breast feeding has been associated with slower gains in infancy weight 4 and body fat 5, and although debated has also been linked to lower risk for obesity and associated metabolic disease risk across the life course 6. It is unclear whether the slower infancy weight gain in breast‐fed babies is a result of lower total calorie intake or related to the nutrient composition of human breast milk (HM). Previous studies documenting HM energy or macronutrient contents have been recently reviewed 7, 8. Most were performed in small sample sizes, and few attempted to assess the influence of HM composition on subsequent infancy growth outcomes.

The evidence on the potential effects of milk nutrient composition on infancy growth is therefore almost entirely limited to trials comparing artificial infant milk formulas. They report that higher milk protein concentrations increase infancy weight gain and predisposition to obesity 9, but there is inconsistent evidence on milk calorie contents 10, 11. In the absence of large population studies, we aimed to investigate the relationships between HM total calorie content, macronutrient contents, or individual lipid species and infancy growth, in a large UK birth cohort study. We hypothesised that specific HM composition may be associated with different patterns of weight and adiposity gain during infancy.

## Patients and methods

### Study design

The Cambridge Baby Growth Study (CBGS) is a prospective birth cohort, focussing on antenatal and early postnatal determinants of infancy growth, as previously described 5. Mothers were recruited during early pregnancy from a single antenatal centre in Cambridge (2001–2009). The whole cohort included 1585 singleton, late preterm/term (gestation ≥36 weeks) infants with measurements at birth, of whom 924 mothers were breast feeding their infants at eight weeks of age. A subcohort of 614 mother–infant dyads, where a breast milk sample was available, is described in the current report. The study was approved by the Cambridge Local Research Ethics Committee, and all mothers gave written informed consent.

### Anthropometry

Infants were measured by trained paediatric research nurses, with weight, length and skinfold thickness assessed in the newborn, and then at three and 12 months of age. Weight was measured to the nearest 1 g using a Seca 757 electronic baby scale (Seca, Birmingham, UK). Supine length was measured to the nearest 0.1 cm using a Seca 416 Infantometer (Seca, Birmingham, UK). BMI was then calculated. Skinfold thickness was measured in triplicate at four sites (triceps, subscapular, flank and quadriceps) on the left hand side of the body using a Holtain Tanner/Whitehouse Skinfold Caliper (Holtain Ltd, Crymych, UK).

### Breast milk collection

To allow for comparable samples and informative macronutrient analysis, mothers who breast‐fed their infants were asked to hand express hindmilk samples, after feeding their infant, between four and eight weeks postnatally, expressing from the same breast that they last used to feed their infant. They repeated this process multiple times, keeping milk samples frozen, and a total of 100 mL of hindmilk was collected over a two‐week period, to reduce within‐day and day‐to‐day variations. Samples were then kept frozen at −20^°^C, until processed at a single time point. The pooled sample was thoroughly mixed before analysis.

Overall, infant feeding practice (exclusive breast vs. mixed feeding) was assessed by questionnaire at age three months, with detailed questions about current feeding, and age at starting supplementary formula milk feeds, as well as completely stopping breast feeding. From this information, infants were categorised as either exclusive breast or mixed feeding at eight weeks of age, contemporaneous with the breast milk collections.

### Breast milk assays

Triglyceride (fat) and lactose (carbohydrate) concentrations were measured in homogenised HM samples using ^1^H‐Nuclear magnetic resonance (NMR) spectra. To determine the lipid concentrations (in mmol/L), 400 microlitres of a homogenised HM sample was mixed with 400 microlitres CDC1_3_ solvent for 10 minutes, and then centrifuged for 30 minutes at 10 000 rpm [Eppendorf centrifuge 5424 (Eppendorf AG, Hamburg, Germany)]. The nonpolar fraction was then used to measure lipid concentrations, from ^1^H‐NMR spectra. Triglyceride concentration was used as a surrogate for total fat content, as this contributes 95–98% of total HM lipid content 12. A further ten lipid species were also quantified: linoleic acid, diglycerides, monoglycerides, docosahexaenoic acid, 18:1/16:1, esterified cholesterol, free cholesterol, total cholesterol, omega 3, monounsaturated fatty acid and polyunsaturated fatty acids, as described previously 13. Lactose, the major HM carbohydrate, was measured from the polar fraction of the milk sample, using ^1^H 1D NOESY spectroscopy. Reproducibility of the NMR methods was assessed: the coefficient of variation (CV) for NMR itself was 0.03–0.3% for lipid analysis, and 0.1–0.6% for analysis of polar metabolites, such as lactose. Analysis of different aliquots from the same sample showed CVs of 0.3–5.8% for lipids and 0.4–4.7% for the polar metabolites. NMR spectral peaks were calibrated using Topspin and analysed based on previous work 14. For protein, total nitrogen was measured by the Dumas method, and the protein factor conversion of 6.25 was used to calculate crude protein content.

Previous work has shown that storage conditions can potentially affect macronutrient content, especially fat content due to the continued activity of lipases and coalescence of fat globules 15. However, we were careful to homogenise the HM samples before analysis. There was no effect of storage time on macronutrient calories, %fat or %carbohydrate; however, %protein was modestly positively associated with the storage time [% per year, B (correlation coefficient) 0.01, p = 0.01]. For this reason, analyses were adjusted for storage time using multiple regression.

### Calculations

Age‐ and sex‐appropriate standard deviation scores (SDS) were calculated for infant weight, length and BMI measurements, adjusting for gestational age in the newborn, by comparison with the UK 1990 growth reference 16, using the LMS Pro software 17. For each of the four skinfold thicknesses, an internal SDS was calculated, adjusted for age, and the mean of the four skinfold SDS was used as estimate measure of adiposity in analyses.

The metabolisable energy content of HM was calculated using Atwater conversions, taking energy contents of 4, 4 and 9 kcal/g for protein, lactose and fat, respectively 18, and HM total calorie content (TCC) was then calculated as kcal/100 mL. The nutrient density method was used to present macronutrient contents as percentages of total calorie content (i.e. %fat, %carbohydrate and %protein) 19. To distinguish independent effects of the individual lipid species investigated, as the lipid species were highly intercorrelated (all Spearman's coefficients >0.51, p < 0.0005), we used a residual nutrient method: each lipid concentration was regressed against the triglyceride concentration, standardised residuals for each lipid species were calculated, and these values were used in subsequent analysis.

### Statistics

The demographics of the cohort subgroup with HM samples were compared to those of the entire CBGS cohort, and in particular to all mother–infant pairs who were breast feeding (either exclusively or mixed feeding) at eight weeks, using *t*‐tests, chi‐squared tests or independent sample median tests.

Relationships between HM TCC, or %macronutrient contents, and infancy growth were investigated, using multivariate regression models, including the following variables: birthweight, gestational age, infant sex, nutrition type and HM storage time. Analyses were performed using SPSS version 20 (IBM Corp., Armonk, NY), and statistical significance was indicated by p value <0.05.

## Results

### Cohort description

The subcohort of 614 mothers of singleton, late preterm/term infants who provided a HM sample was similar to all mother–infant pairs in CBGS who were breast feeding (exclusively or in combination with formula feeding, n = 924). There were no differences with respect to gestational age, maternal age, maternal prepregnancy BMI, maternal primiparity, ethnicity, infant size at birth and subsequent growth to 12 months of age. Further details of the subcohort are shown in Table 1.

**Table 1:** Description of the study members who provided a breast milk sample, in relation to the wider Cambridge Baby Growth Study cohort. Median and IQR are displayed

	Mothers providing HM samples (n = 614)	All CBGS mothers exclusively breast and mixed feeding (n = 924)
Demographics		
Gestational age (weeks)	40.1 (39.1–41.0)	40.0 (39.1–41.0)
Maternal age (years)	33.9 (31.1–36.5)	34.0 (31.2–36.5)
Maternal BMI (kg/m^2^)	22.8 (20.9–25.2)	22.7 (20.8–25.2)
Index of deprivation	9.0 (6.9–9.0)	9.0 (6.8–9.0)
Maternal primiparity (%)	43	42
White caucasian (%)	96	96
Infant sex (% male)	51	51
Exclusive breast feeding (%)	73	77
Growth data		
Birth		
Weight (kg)	3.56 (3.22–3.87)	3.55 (3.22–3.85)
Length (cm)	51.5 (50.0–53.5)	51.5 (50.0–53.3)
Mean skinfold thickness (mm)	6.2 (5.3–7.4)	6.1 (5.2–7.3)
BMI (kg/m^2^)	13.3 (12.2–14.3)	13.3 (12.2–14.2)
3 months		
Weight (kg)	6.10 (5.60–6.64)	6.09 (5.59–6.62)
Length (cm)	61.2 (59.4–63.0)	61.2 (59.5–63.0)
Mean skinfold thickness (mm)	10.8 (9.4–11.9)	10.7 (9.4–11.9)
BMI (kg/m^2^)	16.3 (15.4–17.2)	16.2 (15.3–17.2)
12 months		
Weight (kg)	9.85 (9.10–10.60)	9.88 (9.15–10.60)
Length (cm)	75.8 (74.0–77.7)	75.6 (73.9–77.7)
Mean skinfold thickness (mm)	11.0 (9.8–12.5)	11.0 (9.7–12.4)
BMI (kg/m^2^)	17.1 (16.2–18.0)	17.1 (16.3–18.1)

### Human milk macronutrient contents

For the 614 HM samples analysed, TCC was [median (IQR)] 61.8 (53.7–71.3) kcal/100 mL. The macronutrient composition was as follows: fat (triglycerides) 2.6 (1.7–3.6) g/100 mL; protein 1.2 (1.1–1.2) g/100 mL; and carbohydrate (lactose) 8.6 (8.2–8.8) g/100 mL. Macronutrient contents expressed as calories per 100 mL and percentages of TCC are shown in Table 2.

**Table 2:** Human milk macronutrient contents and their associations with infancy growth (based on n = 614 samples)

	Fat		Carbohydrate		Protein		Total calorie content	
Macronutrient contents^a^								
Calories (kcal) per 100 mL	23.1 (15.4–32.4)		34.3 (32.9–35.3)		4.6 (4.2–5.1)			
%macronutrient content^b^	37.3 (28.4–48.9)		55.2 (47.6–62.9)		7.5 (6.4–9.0)			

Associations with growth	B	p	B	p	B	p	B	p
Weight SDS at 3 month	−0.001	0.7	0.001	0.8	0.02	0.3	−0.002	0.4
Weight SDS at 12 month	−0.005	0.1	0.006	0.2	0.03	0.1	−0.005	0.1
Delta weight SDS 3–12 month	−**0.007**	**0.02**	**0.008**	**0.02**	0.03	0.1	−**0.006**	**0.02**
Mean skinfold SDS at 3 month	−0.004	0.2	0.004	0.1	0.02	0.3	−0.003	0.3
Mean skinfold SDS at 12 month	−**0.009**	**0.001**	**0.01**	**<0.0005**	0.03	0.1	−**0.007**	**0.008**
Delta skinfold SDS 3–12 month	−**0.007**	**0.04**	**0.008**	**0.03**	0.02	0.4	−0.005	0.08
BMI SDS at 3 month	−0.004	0.3	0.004	0.3	0.02	0.4	−0.002	0.4
BMI SDS at 12 month	−**0.01**	**0.002**	**0.01**	**0.002**	**0.04**	**0.04**	−**0.008**	**0.02**
Delta BMI SDS 3–12 month	−**0.01**	**0.005**	**0.01**	**0.005**	0.04	0.08	−**0.008**	**0.01**
Length SDS at 3 month	0.002	0.5	−0.003	0.4	0.01	0.5	−0.001	0.6
Length SDS at 12 month	0.004	0.3	−0.005	0.2	0.003	0.9	0.000	0.9
Delta length SDS 3–12 month	0.001	0.6	−0.003	0.6	−0.005	0.7	0.001	0.6

a Median (IQR).

b %macronutrient was calculated as macronutrient energy/total energy content.

Models were adjusted for exclusive breast vs. mixed feeding at eight weeks, sex, GA, birthweight and duration of sample storage.

Associations with p < 0.05 are highlighted in bold.

HM total calorie and macronutrient contents were unrelated to mother's prepregnancy BMI, pregnancy weight gain, parity, gestational age at delivery or socioeconomic status (assessed using home postcode‐based index of multiple deprivation scores as reported previously 20) and were also unrelated to infant sex (data not shown).

Seventy‐seven percent of the mothers who provided a HM sample were exclusively breast feeding at 8 weeks; the others gave their infants both breast milk and infant formula milk (mixed feeding). HM of exclusively breast feeding mothers contained higher TCC (medians) (62.6 vs. 58.7 kcal/100 mL), higher %fat (37.6 vs. 35.0%), but lower %protein (7.3 vs. 8.3%) and %carbohydrate (54.7 vs. 57.5%), all p < 0.05. All further analyses were adjusted for exclusive breast feeding versus mixed feeding, using multivariate regression modelling.

### Associations with infancy growth

**Figure 1:**
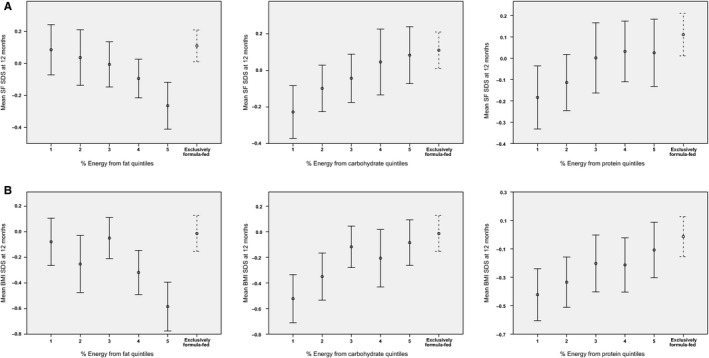
Infant adiposity at 12 months by quintiles of human milk macronutrient contents at 4–8 weeks. (A) 12‐month SF SDS: skinfold standard deviation score as the mean SDS of measurements at four sites. Circles and error bars indicate group means and 95% confidence intervals. (B) 12‐month BMI SDS. Circles and error bars indicate group means and 95% confidence intervals.

As shown in Table 2, HM TCC at 4–8 weeks was inversely associated with BMI (p = 0.02) and adiposity (p = 0.008) at age 12 months, and with three‐ to 12‐month gains in weight (p = 0.02) and BMI (p = 0.01). With regard to %macronutrient contents (Table 2), HM %fat was inversely associated with BMI and adiposity at 12 months, and inversely associated with three‐ to 12‐month gains in weight, BMI and adiposity. In contrast, HM %carbohydrate was positively related to weight, BMI and adiposity gains between three and 12 months. Figure 1 shows that the relationships between quintiles of HM %fat or %carbohydrate and adiposity/BMI at 12 months were broadly linear. HM %protein was positively correlated to BMI at 12 months (p = 0.04), with no association with 12‐month weight or adiposity, or three‐ to 12‐month gains. Figure 1 also shows adiposity/BMI for 271 exclusively formula‐fed CBGS infants at the time of HM sample collection, for comparison. HM %macronutrient contents showed no relationships with infant length at any age.

Sensitivity analyses were carried out, separately by feeding group (exclusive breast feeding vs. mixed feeding at eight weeks). These showed the same directions of associations as in the total population (full data not shown). For example, in the exclusively breast feeding subgroup (n = 389), the associations with 12‐month adiposity were as follows: % HM protein: B 0.02, p = 0.3, % carbohydrate: B 0.009, p = 0.01, % fat: B −0.007, p = 0.02 and total calories: B −0.005, p = 0.07.

### Human milk‐specific lipid species

Concentrations of ten specific HM lipid species are shown in Table 3. In separate models for each lipid species (adjusted for birthweight, gestational age, sex and exclusive breast vs. mixed feeding), all ten lipid species showed inverse associations with infant adiposity at 12 months (data not shown). Using the residual nutrient method, linoleic acid was the only lipid species that remained inversely related to infant adiposity at 12 months (p = 0.05).

**Table 3:** Concentrations of human milk lipid species (mmol/L) n = 614

Lipid species	Median (IQR) mmol/L
Linoleic acid	6.62 (4.39–9.17)
Diglycerides	1.95 (1.19–3.02)
Monoglycerides	0.63 (0.38–0.94)
DHA	0.32 (0.22–0.44)
18:1/16:1	0.77 (0.53–1.08)
Esterified cholesterol	0.18 (0.13–0.24)
Free cholesterol	0.20 (0.14–0.29)
Total cholesterol	0.37 (0.26–0.49)
Omega 3	2.48 (1.62–3.53)
MUFA & PUFA	80.96 (54.66–110.56)

DHA = Docosahexaenoic acid; 18:1/16:1 = Oleic/palmitoleic acid; MUFA and PUFA = Monounsaturated fatty acid and polyunsaturated fatty acid.

## Discussion

To our knowledge, this study of 614 mother–infant pairs is the largest report describing HM macronutrient contents, and the first extensive study to investigate their relationships with infancy growth. We showed inverse associations between HM total calorie content and subsequent gains in weight and BMI, and also later adiposity. Regarding individual HM macronutrients, %carbohydrate was positively correlated to subsequent infant weight, BMI and adiposity gains, whereas %fat was negatively associated with these infancy outcomes. HM %protein was weakly positively associated with BMI at 12 months but not gains in adiposity.

Associations between HM contents and infancy growth have not been previously reported, largely due to the lack of other large studies. However, in support of our study design, the observed positive association between HM %protein content and 12‐month BMI is consistent with experimental evidence from large clinical trials that tested isocaloric infant milk formulas containing high versus usual protein contents. Unfortunately, HM intakes were not assessed in our study and therefore we cannot assess whether the associations observed with HM contents were mediated by nutrient intakes.

Of relevance, a recent study reported an inverse association between fat intake at two years of age and body fat, assessed by bioelectrical impedance analysis at 20 years 21, also suggesting that early diet containing greater fat may benefit later body composition, either directly or indirectly. A higher proportion of ingested carbohydrate may promote storage of glycogen and fat. Alternatively, it is possible that infants fed HM with lower %fat may feel less satiated and drink larger volumes of milk, hence gaining more weight. This hypothesis is supported by previous observations that HM %fat was inversely related to the volume of HM intake, whereas %lactose was positively correlated 22, and by older studies reporting that infants consuming formula milk with lower energy had higher dietary intakes 11.

A recent systematic review concluded that higher protein intake in infancy and early childhood is associated with faster weight gain and greater BMI in childhood 23. We did not have further detailed body composition data, making it difficult to distinguish between gains in lean mass or fat mass. We found relatively less interperson variability in %protein than in other macronutrients, and it may be that larger differences in %protein, such as those seen in formula milk studies 9, are needed to observe significant influences of protein content on infancy weight gain.

It is difficult to directly compare our results with other previous studies of HM constituents, due to differences in the timing of HM collection with pooling of samples, sampling of solely hindmilk, HM assays and the nature of the populations sampled. A recent systematic review summarised the results of ‘mature’ HM samples (taken 2–4 weeks postnatally) 7, a total of the following: 415 for protein, 476 for carbohydrate and 567 for lipids, pooling data from a minimum of 18 studies worldwide, with the largest sample size of 71 in any single study. Our study is therefore far larger than any other reported, allowing informative associations with HM macronutrient contents in the range of the previously pooled meta‐analysed values.

Heinig et al. 24 showed that total energy and protein intakes were positively correlated to weight, not only in formula‐fed infants (n = 46) but also in those exclusively breast‐fed (n = 73). Specifically, total protein intake was positively correlated with three‐ to six‐month and six‐ to nine‐month weight gain in breast‐fed infants. Butte et al. 25 reported that intakes of HM protein, fat and carbohydrate were all positively correlated with weight gain and fat‐free mass gain (assessed using a multicomponent body composition model) at 3–6 months, but not with fat mass gain, in 40 breast‐fed infants and 36 formula‐fed infants. These studies assessed intakes, not HM content, and were also much smaller cohorts, assessing anthropometry at different time points, with different methods for HM collection and nutrient analysis. Further, larger studies, across different populations, with information on both composition and intakes, are needed, using a standardised sampling protocol.

It is interesting to note that the associations between HM macronutrient contents and infant anthropometry in our study were mainly with weight, BMI and adiposity, with no apparent influence on length gains. This is surprising as weight gain and statural growth are closely linked in infancy; hence, it may be speculated that other confounders could explain the findings with adiposity. Maternal characteristics could be one source of confounding. Some small previous reports have shown correlations between specific maternal factors and HM fat content including parity 26 and maternal anthropometric status 27. However, these associations have not been extended to all macronutrients or been well replicated, and we found no associations between such maternal factors and HM nutrient contents. We did not assess maternal diet, but other studies have reported no relationship with HM contents 27.

Alternatively, it may be that other constituents in breast milk, such as individual lipid moieties, could explain the relationships seen with HM macronutrients and in particular the inverse relationships between lipid and infancy adiposity. Disentangling the potential independent contribution to growth from individual fatty acids, which are highly correlated with total lipid, proved to be difficult. Only the omega‐6 fatty acid, linoleic acid showed a consistent independent inverse relationship with later infancy adiposity. Of note, in the literature, n‐3 and n‐6 long‐chain polyunsaturated fatty acids have received interest with respect to growth and development, with for example suggested beneficial effects on growth with alpha‐linoleic/DHA supplementation in developing countries 28; however, generally there is an overall paucity of data for n‐3 or n‐6 LC‐PUFAs 15, 16, 17. Further detailed LC‐PUFA analyses and subsequent studies are required to confirm our finding and investigate this area further.

The higher total calorie content found in HM from mothers who were exclusively breast feeding, when compared to those mixed feeding, is consistent with other observations, maintaining sufficient continued nutrition 29, and suggesting that HM energy content may be downregulated by infants mixed feeding. The higher %fat, with lower %protein and %carbohydrate, seen in milk of mothers exclusively breast feeding, may support our findings of growth associations in indicating that this is a beneficial HM composition with regard to subsequent infant adiposity. It could also be speculated that a higher %fat results in greater infant satiation, resulting in continued breast feeding, whereas hungrier babies consuming HM with lower fat content are more likely to be given supplementary formula milk.

Alternatively, there may be differences in HM production, regulated by the suckling infant, or even potential confounding by the collection techniques used by mothers expressing milk. Hindmilk contains more fat than foremilk 30; therefore, it is not implausible that the exclusively fed infants consumed more milk, and their HM samples contained relatively more hindmilk. We adjusted for exclusive breast versus mixed feeding in our subsequent analytical models, with no interaction seen between feeding type and macronutrient content in analyses, and thus, this issue is unlikely to confound the associations with infant growth. Similar trends between HM macronutrient contents and infancy body size/growth were also apparent in the exclusively breast‐fed subgroup: although generally less significant in this smaller group, correlations were in the same direction, and with similar effect sizes.

Limitations of our study include the lack of information on HM intakes, and therefore, it was not possible to calculate total energy and macronutrient intakes. HM lipid and protein contents are known to vary between individual feeds and with different stages of lactation 7, 8. Mothers were encouraged to pool, over a period of two weeks, their collections of expressed hindmilk; however, it is possible that systematic differences existed between collections and information on the timings of milk collection were not recorded. Some of these limitations will be tackled and subject of further studies.

### Conclusion

In conclusion, in this large study of HM macronutrient content, we found that HM nutrient composition in early infancy differs between exclusively breast feeding and mixed feeding mothers. Of note, HM %fat and %carbohydrate predicted changes in infancy weight and adiposity gains up to age 12 months, with %protein positively related to 12‐month BMI. There were no associations with length gains. Although data on milk intakes were unavailable, our findings suggest that higher HM %fat but lower %carbohydrate may be associated with lower gains in adiposity and BMI.

## Funding

PP was supported by a MRC Clinical Training Fellowship (G1001995). The Cambridge Baby Growth Study has been supported by the European Union, the World Cancer Research Foundation International, the Medical Research Council, the NIHR Cambridge Comprehensive Biomedical Research Centre, the Newlife Foundation for disabled children, the Mothercare Group Foundation and Mead Johnson Nutrition.

## Conflict of interest statement

This study received unconditional funding support from Mead Johnson Nutrition. MH Schoemaker and EAF van Tol are employees of Mead Johnson Nutrition. No other authors declare a conflict of interest.
